# Supplementary material for: Reconciling Mining with the Conservation of Cave Biodiversity: A Quantitative Baseline to Help Establish Conservation Priorities
Source: PLoS One. 2016 Dec 20;11(12):e0168348. doi: 10.1371/journal.pone.0168348 (PMC5173368; doi:10.1371/journal.pone.0168348)
Supplement: S1 Dataset — (ZIP) [file pone.0168348.s002.zip › Taxa/Serra Norte/SN_2013/N4WS/Lista N4WS-15.pdf]

CAVIDADE N4WS-0015

Classe	Ordem	Fam/Outros	Gên/Outros	Espécie	S-U	U-S	Zon.
Annelida	Oligochaeta			jovem	2	14	E/P/A
Arachnida	Acari	(Oribatida)		sp.3		X	E
Arachnida	Acari	(Parasitengonina)		sp.2		X	E
Arachnida	Acari	(Parasitengonina)		sp.3		X	E/P/A
Arachnida	Acari	(Parasitengonina)		sp.13		X	P/A
Arachnida	Acari	Argasidae	*Ornithodoros*	sp.1	X	X	E/A
Arachnida	Acari	Holothyrida		jovem	X		
Arachnida	Acari	Laelapidae		sp.1		X	A
Arachnida	Acari	Macronyssidae		sp.1		X	P
Arachnida	Acari	Neothyridae	*Diplothyrus*	sp.1		X	E
Arachnida	Acari	Trombiculidae		sp.	X		
Arachnida	Acari	Trombidioidea		sp.	X		
Arachnida	Amblypygi	Charinidae	*Charinus*	sp.1	X	8	P/A
Arachnida	Amblypygi	Phrynidae	*Heterophrynus*	*longicornis*	X	1	A
Arachnida	Araneae	Araneidae	*Alpaida*	aff. *negro*		X	E
Arachnida	Araneae	Araneidae	*Micrathena*	sp.1	X		
Arachnida	Araneae	Caponiidae		sp.1		X	P/A
Arachnida	Araneae	Corinnidae	*Creugas*	sp.1	1	22	A
Arachnida	Araneae	Ctenidae		jovem	4	4	E/P
Arachnida	Araneae	Drymusidae	*Drymusa*	*spelunca*	X	X	P/A
Arachnida	Araneae	Filistatidae		sp.1	X		
Arachnida	Araneae	Ochyroceratidae	*Ochyrocera*	sp.1	X	X	E/P/A
Arachnida	Araneae	Ochyroceratidae	*Speocera*	sp.1	X	X	E/P/A
Arachnida	Araneae	Ochyroceratidae	*Theotima*	sp.	X		
Arachnida	Araneae	Pholcidae	*Leptopholcus*	sp.1		X	E/P
Arachnida	Araneae	Pholcidae	*Mesabolivar*	sp.	X		
Arachnida	Araneae	Pholcidae	*Metagonia*	sp.	X		
Arachnida	Araneae	Pholcidae	Ninetinae	sp.1		X	P
Arachnida	Araneae	Salticidae		sp.	X	*X*	
Arachnida	Araneae	Scytodidae	*Scytodes*	*eleonorae*	2		
Arachnida	Araneae	Scytodidae	*Scytodes*	*globula*		32	E/P/A
Arachnida	Araneae	Segestriidae		jovem	X		
Arachnida	Araneae	Tetrablemmidae	*Matta*	sp.1		X	P/A
Arachnida	Araneae	Theraphosidae	aff. *Holothele*	sp.1	X	9	P
Arachnida	Araneae	Theridiidae	*Theridion*	sp.1		X	E/P/A
Arachnida	Araneae	Theridiosomatidae	*Plato*	sp.1	X	X	E/P/A
Arachnida	Opiliones	Escadabiidae		sp.2	X	X	P/A
Arachnida	Opiliones	Stygnidae	*Protimesius*	*gracilis*		1	P
Arachnida	Palpigradi			jovem	X	X	P
Arachnida	Pseudoscorpiones	Bochicidae		sp.	X		
Arachnida	Pseudoscorpiones	Chernetidae	*Spelaeochernes*	sp.1	X	X	P/A
Arachnida	Pseudoscorpiones	Chthoniidae	*Pseudochthonius*	sp.1	X	X	E/P/A
Arachnida	Schizomida	Hubbardiidae		jovem		X	P
Chilopoda	Geophilomorpha			sp.	X		
Chilopoda	Scolopendromorpha	Scolopocryptopidae	*Tidops*	sp.1		1	P
Chilopoda	Scutigeromorpha	Pselliodidae	*Sphendononema*	*guildingii*	X	4	E/A
Diplopoda	Polydesmida	Fuhrmannodesmidae		jovem		X	P
Diplopoda	Polydesmida	Pyrgodesmidae		sp.1	X	X	
Diplopoda	Polyxenida	Polyxenidae		sp.1		X	E/P/A

Diplopoda	Spirostreptida	Pseudonannolenidae	*Pseudonannolene*	jovem	X	1	P
Entognatha	Collembola	Cyphoderidae		sp.1		X	P/A
Entognatha	Collembola	Isotomidae		sp.1		X	P/A
Entognatha	Collembola	Paronellidae		sp.1		X	E/P/A
Entognatha	Collembola	Paronellidae		sp.4		X	E/P/A
Entognatha	Collembola	Sminthuroidea		sp.1	X	X	A
Entognatha	Collembola	Sminthuroidea		sp.2	X	X	P/A
Entognatha	Diplura	Campodeidae		sp.1		X	P
Entognatha	Diplura	Japygidae		sp.3		X	A
Insecta	Blattodea	Blaberidae		sp.1	5	1	A
Insecta	Blattodea	Blattidae		sp.1		1	P
Insecta	Coleoptera	Carabidae		sp.19	X	X	E
Insecta	Coleoptera	Elateridae		sp.	X		
Insecta	Coleoptera	Histeridae		sp.4	X		
Insecta	Coleoptera	Hydrophilidae		jovem		X	A
Insecta	Coleoptera	Staphylinidae		sp.63		X	A
Insecta	Coleoptera	Tenebrionidae		jovem	X		
Insecta	Diptera	Anthomyiidae		sp.	X		
Insecta	Diptera	Cecidomyiidae	Cecidomyiinae	sp.	X	X	P
Insecta	Diptera	Chironomidae		sp.		X	E/A
Insecta	Diptera	Chloropidae		sp.		X	E/P/A
Insecta	Diptera	Drosophilidae	*Drosophila*	*eleonorae*		X	E/P/A
Insecta	Diptera	Drosophilidae	*Drosophila*	sp.	X		
Insecta	Diptera	Ephydridae		sp.		X	A
Insecta	Diptera	Milichiidae		sp.	X		
Insecta	Diptera	Muscidae		jovem		X	E
Insecta	Diptera	Phoridae	Metopininae	sp.		X	E/A
Insecta	Diptera	Phoridae	Phorinae	sp.		X	A
Insecta	Diptera	Psychodidae	*Breviscapus*	sp.		X	P/A
Insecta	Diptera	Psychodidae	*Edentomyia*	*piauiensis*	X		
Insecta	Diptera	Psychodidae	Phlebotominae	sp.		X	P/A
Insecta	Diptera	Psychodidae	*Pintomyia*	*gruta*	X		
Insecta	Diptera	Psychodidae	*Telmatoscopus*	sp.		X	A
Insecta	Diptera	Sciaridae		jovem		X	A
Insecta	Diptera	Streblidae	*Trichobius*	sp.		X	A
Insecta	Diptera	Tipulidae		sp.	X		
Insecta	Heteroptera	Cydnidae		sp.	X	X	P/A
Insecta	Heteroptera	Dipsocoroidea		sp.		X	A
Insecta	Heteroptera	Lygaeidae		sp.		X	E
Insecta	Heteroptera	Reduviidae	Emesinae	sp.	X	2	P
Insecta	Homoptera	Cixiidae		sp.1	X	X	E
Insecta	Hymenoptera	Bethylidae		sp.2	X	X	P
Insecta	Hymenoptera	Chalcidoidea		sp.1		X	P
Insecta	Hymenoptera	Diapriidae		sp.1		X	P/A
Insecta	Hymenoptera	Formicidae	Acromyrmex	sp.1	X		
Insecta	Hymenoptera	Formicidae	Camponotus	*atriceps*	X		
Insecta	Hymenoptera	Formicidae	*Hypoponera*	sp.1	X	X	E/A
Insecta	Hymenoptera	Formicidae	Myrmicinae	sp.1		X	P
Insecta	Hymenoptera	Formicidae	*Pachycondyla*	*constricta*	X		
Insecta	Hymenoptera	Formicidae	*Pachycondyla*	sp.2		X	E
Insecta	Hymenoptera	Formicidae	*Paratrechina*	sp.1		X	P
Insecta	Hymenoptera	Formicidae	*Solenopsis*	sp.1	X	X	E/P/A

Insecta	Hymenoptera	Formicidae	*Trachymyrmex*	sp.1		X	E
Insecta	Hymenoptera	Scelionidae		sp.	X		
Insecta	Isoptera	Termitidae	*Nasutitermes*	sp.	X	X	E/P/A
Insecta	Lepidoptera	Galacticidae		sp.		X	A
Insecta	Lepidoptera	Noctuoidea		sp.		1	A
Insecta	Lepidoptera	Tineoidea		sp.	X	X	E/P/A
Insecta	Neuroptera	Myrmeleontidae		jovem	X		
Insecta	Orthoptera	Phalangopsidae	*Phalangopsis*	sp.	X	15	E/P/A
Insecta	Plecoptera			jovem		1	P
Insecta	Psocoptera	Psocomorpha		jovem	X		
Insecta	Psocoptera	Psyllipsocidae		jovem		X	P
Insecta	Thysanura	Nicoletiidae		sp.1	X	X	E
Malacostraca	Isopoda	Armadillidae		sp.1	X	X	E/P/A
Malacostraca	Isopoda	Philosciidae		sp.1		X	E
Malacostraca	Isopoda	Philosciidae		sp.2		X	E
Malacostraca	Isopoda	Scleropactidae	*Circoniscus*	*buckupi*		X	P/A
Nematoda				sp.1		X	P/A
Symphyla		Scutigerellidae	*Hanseniella*	sp.	X	X	
Mammalia	Chiroptera	Furipteridae	*Furipterus*	*horrens*		170	E/P/A
Mammalia	Chiroptera	Phyllostomidae	*Carollia*	*perspicillata*		270	E/P/A
Mammalia	Chiroptera	Phyllostomidae	*Lampronycteris*	*brachyotis*		X	E/P/A
Mammalia	Chiroptera	Phyllostomidae	*Lonchorrhina*	sp.		X	E/P/A
Mammalia	Marsupialia			sp.	1		E
